# Quality of care in Belgian general practices during the COVID-19 pandemic: results of the cross-sectional PRICOV-19 study

**DOI:** 10.1186/s12875-024-02305-8

**Published:** 2024-03-05

**Authors:** Esther Van Poel, Pierre Vanden Bussche, Benoît Pétré, Cécile Ponsar, Claire Collins, Michel De Jonghe, Anne-Françoise Donneau, Nicolas Gillain, Michèle Guillaume, Sara Willems

**Affiliations:** 1 Department of Public Health and Primary Care, Ghent University, Ghent, Belgium; 2 Quality and Safety Ghent, Department of Public Health and Primary Care, Ghent University, Ghent, Belgium; 3 Department of Public Health, Faculty of Medicine, University of Liège, Liège, Belgium; 4 Institute of Health and Society, University of Louvain, Louvain, Belgium; 5 Research Centre, Irish College of General Practitioners, Dublin, D02 XR68 Ireland; 6 Centre Académique de Médecine Générale, Université Catholique de Louvain, Brussels, Belgium

**Keywords:** Primary health care, General practice, Quality of care, Equity, Patient safety, Timeliness, Person-centeredness, Efficiency, Effectiveness, COVID-19, PRICOV-19

## Abstract

**Background:**

The COVID-19 pandemic immensely impacted care provision, including quality of care in general practice. This paper aimed: (1) to assess how Belgian general practices acted upon the six dimensions of quality of care during COVID-19; (2) to study differences between the three Belgian regions; and (3) to benchmark the performance of the Belgian practices against the performance in other European countries.

**Methods:**

The data collected from 479 Belgian practices during 2020–2021 using an online survey as part of the international cross-sectional PRICOV-19 study were analyzed.

Hereby, descriptive statistics, chi-squared tests, and binary logistic regression analyses were performed. Thirty-four survey questions related to the six dimensions of quality of care were selected as outcome variables. The adjusted regression models included four practice characteristics as covariates: practice type, being a teaching practice for GP trainees, multidisciplinarity of the team, and payment system.

**Results:**

Belgian practices made important organizational changes to deliver high-quality care during COVID-19. Most practices (*n* = 259; 56.1%) actively reached out to vulnerable patients. Limitations to the practice building or infrastructure threatened high-quality care in 266 practices (55.5%). Infection prevention measures could not always be implemented during COVID-19, such as using a cleaning protocol (*n* = 265; 57.2%) and providing a separate doctor bag for infection-related home visits (*n* = 130; 27.9%). Three hundred and sixty practices (82.0%) reported at least one safety incident related to a delayed care process in patients with an urgent condition. The adjusted regression analysis showed limited significant differences between the Belgian regions regarding the quality of care delivered. Belgian practices demonstrated varied performance compared to other European countries. For example, they excelled in always checking the feasibility of isolation at home but reported more patient safety incidents related to timely care than at least three-quarters of the other European countries.

**Conclusions:**

Future studies using different design methods are crucial to investigate which country and practice characteristics are associated with delivering high-quality care.

**Supplementary Information:**

The online version contains supplementary material available at 10.1186/s12875-024-02305-8.

## Background

The COVID-19 pandemic profoundly impacted healthcare services globally, including primary care (PC) [1]. General practitioners (GPs), who serve as the first point of contact for potentially infected patients and provide both short- and long-term follow-up care for most patients [2, 3], were faced with unprecedented structural and organizational challenges to deliver high-quality care [4]. These included amended tasks like performing teleconsultations, increased collaboration with secondary care services and neighboring general practices (GP practices), and limited resources [5–8]. In the initial months of the pandemic, the lack of established protocols for COVID-19 and the continued management of non-COVID patients in an exceptional context was particularly challenging [9]. Later, healthcare providers were overwhelmed with guidelines which quickly changed [10]. In Belgium, guidelines for safe care during COVID-19 were developed by several organizations. For example, the professional organization Domus Medica provided guidelines for safe practice in Flanders [11], while its counterpart SSMG (la Société Scientifique de Médecine Générale) was responsible for drawing guidelines in Wallonia [12]. At the national level, guidelines were also established through the health institute Sciensano in collaboration with policymakers, scientists, and healthcare professionals [13]. Previous Belgian research demonstrated that GPs felt burdened and strained by the need to stay constantly informed [14].

Additionally, Belgian GPs were worried about unmet healthcare needs among patients: there was a temporary ban on all planned non-urgent care in PC and secondary care, including chronic and preventive care, while also patients postponed care because of fearing infection or assuming that GPs were overwhelmed [15]. Furthermore, the pandemic led to an increased number of patients with greater vulnerability [16, 17], including chronic patients for whom COVID-19 posed a potentially life-threatening risk and those who were indirectly susceptible to health problems due to public health and social measures, such as patients with a limited social network or already precarious mental health [18, 19]. GPs were crucial in identifying these vulnerable patients and limiting collateral damage [5, 15, 20].

Given that GP practices were urged to reorganize their way of working and revise care processes in unprecedented circumstances [21], delivering high-quality care became an important challenge during COVID-19 [22, 23]. This paper has three aims: (i) to describe the organization of care in Belgian GP practices from the perspective of the six dimensions of quality of care: equity, patient-centeredness, safety, effectiveness, timeliness, and efficiency; (ii) to investigate the differences in terms of quality of care during COVID-19 between the three Belgian regions (i.e., the Flemish Region (FR), Brussels-Capital Region (BCR), and Walloon Region (WR)); and finally, (iii) to benchmark the Belgian performance on the six dimensions of quality of care against the performance in 36 other European countries. The findings could serve as an initial step towards formulating policies for potential future outbreaks where there is an increased number of patients with symptoms of infections within GP practices.

## Methods

### Study design and setting

During the summer of 2020, an international consortium comprising over 45 European research institutes was established under the coordination of Ghent University (Belgium) to launch the PRICOV-19 study [24]. This multi-country cross-sectional study aimed to investigate various aspects related to the organization of GP practices during the COVID-19 pandemic, including the provision of high-quality care, changes in task roles, the impact on the well-being of healthcare providers, and potential differences among various types of practices and healthcare systems. Data were collected in 37 European countries and Israel. For Belgium, data collection took place in all three regions. The FR is located in northern Belgium and predominantly Dutch-speaking, while the WR in the south is primarily French-speaking. The BCR, the capital of Belgium, operates as a separate bilingual region, officially recognizing Dutch and French as its languages.

### Measurement

Data were collected using an online survey among GP practices. The survey was developed at Ghent University in multiple phases, including a pilot study among 159 practices in Flanders (Belgium). Details on the study protocol and the survey’s development are published elsewhere [24]. The final survey included 53 items divided into six sections: patient flow; infection prevention; information processing; communication; collaboration and wellbeing; and practice and participant characteristics. This version was translated into Dutch and French using the forward-backwards method. The REDCap platform was used to host the survey [25].

### Sampling and recruitment

In each country, GP practices were selected following a pre-defined recruitment procedure, preferably random. Only one survey was completed per practice, usually by a GP. Data were collected between November 2020 and December 2021. Belgian practices were recruited between December 2020 and August 2021. A random selection of 1,477 Belgian practices was made from an updated listing on the web application of the National Institute for Health and Disability Insurance (INAMI/RIZIV), which specifically included GPs active as of November 1, 2020 [26]. Being qualified as a GP before 1980 was considered an exclusion criterion to exclude retired GPs or GPs seeing only a limited number of patients. The practices of all selected GPs were invited to participate in the study using a standardized procedure, including several attempts of contact via telephone and email. This resulted in the participation of 370 practices (response rate of 25.1%). An additional convenience sample of 134 GP practices was drawn through the professional and personal networks of the research teams involved. A total of 109 GP practices participated in the study (response rate of 81.3%).

### Data analysis

Data cleaning and statistical analysis were performed at Ghent University using SPSS Statistics for Windows, version 28.0 (IBM Corp., Armonk, N.Y., USA). Data cleaning involved invalidating answer options of ‘I do not know,’ ‘not applicable,’ and ‘no answer.’ Additionally, potential outliers in the dataset were checked with the respective country coordinators. In total, 34 quality of care-related items were selected as the outcome variables based on the definitions of the Institute of Medicine regarding the six dimensions [27]: person-centeredness and equity (#15), safety and effectiveness (#13), timeliness (#4), and efficiency (#2). Their respective survey items, original and recoded answer options, and number of missing values are included in Additional File (1) Based on a literature review [28, 29], four practice characteristics were used as covariates: practice type (solo, duo, or group practice based on the number of GPs in practice); being a teaching practice for GP trainees (yes or no); payment system (fee-for-service or capitation); and multidisciplinarity of the team (having at least one other paramedical discipline working in practice apart from a GP or not having such). Frequencies and valid percentages were used to describe the outcome variables and four practice characteristics. Given the lack of publicly available data on the distribution of GP practice among the Belgian regions, an assessment of its representativeness is made based on the number of GPs in Belgium. Differences in practice characteristics between the regions were analyzed using Pearson’s chi-squared tests. Unadjusted and adjusted binary logistic regression models were used to examine the differences between the regions on the outcome variables. A comparison between both models is included in Additional File (2) Odds ratios and 95% confidence interval were reported. The criterion of statistical significance (two-fold, p) was determined at 0.05. Furthermore, countries were first ranged using the valid percentages to benchmark the Belgian performance on each outcome variable against the performance in the other 36 participating European countries. Next, quartiles were calculated (Q1-Q4), where Q1 included the eight countries that performed best on a given outcome variable, and Q4 referred to the eight countries with the lowest performance.

## Results

### Description of the participating Belgian GP practices

The characteristics of the 479 Belgian practices included in the cleaned dataset are shown in Table 1. Two hundred and eighty practices were located in the FR (58.5%), 152 (31.7%) in the WR, and 47 (9.8%) in the BCR. Based on the available data on the number of GPs, a representative sample regarding its distribution between the Belgian regions participated in this study. In the FR, the practices were significantly more often duo or group practices compared to the WR and BCR (*p* < 0.001). Also, multidisciplinary practices were significantly more frequent in the FR than in the WR or BCR (*p* < 0.05). No significant differences between the regions were found regarding being a teaching practice for GP trainees or the practice’s payment system.

**Table 1:** Practice characteristics of the participating Belgian GP practices and a comparison between the Belgian regions: descriptive statistics and chi-square tests

		Belgium (total)^b^	Brussels-Capital Region	Walloon Region	Flemish Region	*p* value
GPs^a^	Total population	11,767 (100)	1178 (10.0)	3784 (32.2)	6805 (57.8)	
GP practices	Study sample	479 (100)	47 (9.8)	152 (31.7)	280 (58.5)	
Practice type	Solo	178 (37.3)	24 (52.2%)	88 (58.7)	65 (23.2)	< 0.001
	Duo	93 (19.5)	6 (13.0%)	28 (18.7)	59 (21.1)	
	Group	206 (43.2)	16 (34.8%)	34 (22.7)	156 (55.7)	
Multidisciplinarity of the team	Yes	145 (31.2)	14 (29.8%)	32 (22.2)	98 (35.9)	0.016
	No	320 (68.8)	33 (70.2%)	112 (77.8)	175 (64.1)	
GP trainee teaching practice	Yes	201 (42.3)	17 (37.0%)	63 (42.3)	121 (43.4)	0.717
	No	274 (57.7)	29 (63.0%)	86 (57.7)	158 (56.6)	
Payment system	Fee-for-service	435 (91.0)	39 (83.0)	142 (94.0)	253 (90.7)	0.066
	Capitation	43 (9.0)	8 (17.0)	9 (6.0)	26 (9.3)	

Data are given as n (valid percentage)

*GPs* general practitioners

^a^Information regarding the number of GPs in 2020 is obtained from the IMA Atlas via the following link: http://www.ima-aim.be

^b^One GP practice could not be classified into one of the regions as the zip code was missing

### Performance of the Belgian GP practices

#### Person-centered and equitable care

Table 2 shows the efforts of the practices in delivering person-centered and equitable care during COVID-19. These include extracting a list of patients with a chronic condition at least once from the electronic medical record (*n* = 87; 19.6%) or performing proactive telephone calls to patients with a chronic condition (*n* = 228; 50.2%), a psychological vulnerability (*n* = 161; 35.6%), or known domestic violence problems or issues related to raising or parenting a child (*n* = 63; 14.9%). Practices from the BCR significantly more often reached out to the latter than practices in the WR (*p* < 0.05). In 221 practices (50.2%), GPs and/or GP trainees were more involved during COVID-19 in reaching out to patients who might have postponed health care, and in 102 practices (42.3%), this was the case for non-GP staff members. The involvement of non-GPs in outreach occurred more frequently in the FR than in the WR (*p* < 0.05). In 64.6% of the practices (*n* = 153), non-GP staff members were more involved in providing additional information or explanation to vulnerable patients such as those with low health literacy compared to before COVID-19.

Multilingual communication was available in 9.5% (*n* = 42) of the Belgian practices on their answering machine, 13.5% (*n* = 47) for the practice website, 13.1% (*n* = 36) for the practice leaflet, and 25.1% (*n* = 57) for a COVID-19 leaflet. As expected due to the official bilingualism in the BCR, multilingual communication was more prevalent in BCR practices compared to the WR (*p* < 0.001). The BCR and FR also differed significantly, including a greater focus on multilingualism in the BCR. However, these differences were less pronounced for the answering machine (*p* < 0.01) or the COVID-related leaflet (*p* < 0.01). Finally, a multilingual COVID-related leaflet was more often available in the FR than in the WR (*p* < 0.01).

Practices were asked to which extent they actively checked the patients’ social context. When quarantine or isolation was indicated, one hundred fifty Belgian practices (32.1%) always checked whether this was feasible at home for the patient. In case of a referral to another facility (e.g., getting a COVID-test), 43.4% of practices (*n* = 200) checked whether the transport to the other facility was feasible for the patient. This was significantly less often checked in the FR than in the WR (*p* < 0.05). Compared to before COVID-19, 40.9% of the practices (*n* = 186) checked more or much more frequently whether patients experienced financial problems. The latter was occurred significantly more often in practices in the WR than in the FR (*p* < 0.05). Also, 17.1% of the practices (*n* = 78) checked more or much more frequently whether the patient had experienced domestic violence during COVID-19.

As shown in Table 2, the performance of the Belgian practices to deliver person-centered and equitable care compared to 36 other European countries differed according to the outcome variable. For example, Belgium was in the first quartile for increased screening of domestic or financial issues in patients during COVID-19, while it ranked in the lowest quartile (Q4) for greater involvement of non-GP staff in outreach work, compared to before COVID-19.

#### Safe and effective care

Two hundred sixty-six practices (55.5%) indicated that they experienced limitations to the practice building or infrastructure to deliver high-quality care (Table 2), which was more common in the FR than in the WR (*p* < 0.01). More than one in three (*n* = 176; 37.1%) practices considered making future adjustments to the practice or infrastructure.

In 81.5% of the practices (*n* = 224), patients wishing to make an appointment were informed about symptoms that might prevent them from entering the practice. This happened more frequently in the FR than in the WR (*p* < 0.001) or BCR (*p* < 0.05). Also, in most practices, patients needed to give a reason for the encounter when making the appointment by phone (*n* = 387; 85.4%), in contrast to 68.2% (*n* = 191) when making an online appointment. In 68.7% of the practices (*n* = 314), a protocol was available for answering calls from potential COVID-patients. However, only 26.3% of these practices (*n* = 81) always used such a protocol. Overall, 28.0% of the practices (*n* = 119) always called patients with an online appointment to check their infection risk if this was unclear. In 79.8% of the practices (*n* = 225), a GP was always available as a backup in case of questions when a non-GP answered telephone calls from patients. This was significantly more likely the case in the FR than in the BCR (*p* < 0.01). In 75.2% of the practices (*n* = 319), the most recent information on referring patients to a triage station was within immediate reach in every GP consultation room.

During COVID-19, cleaning employees in more than half of the practices (*n* = 265; 57.2%) always used a detailed protocol for cleaning the practice. In addition, in only 38.5% of the practices (*n* = 178), there was always sufficient time between every consultation to disinfect the consultation room. A total of 27.9% of the practices (*n* = 130) had a separate doctor bag available for infection-related home visits as an infection prevention measure. Belgium’s performance on a European level showed considerable variation, generally ranking in the top half (Q1-Q2) for aspects such as experienced limitations related to the building/infrastructure and showing informative messages in the online appointment system, but positioned lower (Q3-Q4) in areas such as the availability of a telephone protocol and consistently calling online-booked patients to verify the infection risk.

#### Timely care

Regarding the timeliness of care, 310 practices (70.9%) reported a safety incident in which a patient with an urgent condition was seen late because the patient did not come to the practice sooner (Table 2). Almost two-fifths (*n* = 151; 39.3%) of practices encountered similar patient safety incidents because a patient did not know how to reach the GP sooner, which was significantly more common in the BCR (*p* < 0.05) and the FR (*p* < 0.001) than in the WR. About one-fourth (*n* = 105; 26.0%) of the practices experienced at least one safety incident in which a patient with an urgent condition was seen late due to misjudgment as non-urgent during the telephone triage. Almost half of the practices (*n* = 203; 47.9%) experienced a safety incident in which a patient with a fever due to a non-COVID-infection was seen late due to the COVID-19 protocol. All these situations were more often reported in Belgium than in at least three-quarters of the other European countries (Q4).

#### Efficient care

Compared to before COVID-19, non-GP staff members were more involved in triaging patients (*n* = 226; 91.1%) and giving information and recommendations to patients contacting the practice by phone (*n* = 210; 85.4%) (Table 2). Regarding triaging patients, Belgium was ranked in the top of European countries (Q1), where it was in Q3 for the latter outcome variable.

**Table 2:** Comparison of Belgium to 36 other European countries and between the Belgian regions: descriptive statistics and adjusted binary logistic regression models for the six dimensions of quality of care

	Valid percentage				Odds Ratio (95% Confidence Interval)		
	Belgium (total) Quartile	Brussels-Capital Region (BCR)	Walloon Region (WR)	Flemish Region (FR)	BCR vs. WR Reference cat: WR	FR vs. WR Reference cat: WR	BCR vs. FR Reference cat: FR
*Person-centered and equitable care*							
The practice extracted a list of at least one group of patients with a chronic disorder from the electronic medical record system. (yes)	19.6% Q3	28.6%	17.1%	19.5%	1.62 (0.65–4.04)	1.05 (0.56–1.98)	1.54 (0.65–3.66)
The practice actively reached out to …							
patients with a chronic condition who needed follow-up care (yes)	50.2% Q3	55.8%	43.7%	52.6%	1.53 (0.72–3.25)	1.03 (0.64–1.67)	1.48 (0.72–3.05)
psychologically vulnerable patients (yes)	35.6% Q2	37.8%	31.9%	37%	1.02 (0.46–2.24)	0.81 (0.48–1.48)	1.26 (0.60–2.66)
patients with known problems of domestic violence or families with a known problematic parenting situation (yes)	14.9% Q2	27.5%	10.2%	15.5%	2.78* (1.04–7.41)	1.15 (0.55–2.40)	2.42 (0.99–5.90)
Change of roles compared to before COVID-19, including a greater involvement of…							
GP or GP trainees: actively reaching out to patients that might postpone healthcare (yes)	50.2% Q2	53.3%	56.8%	45.6%	0.84 (0.42–1.69)	0.65 (0.41–1.04)	1.29 (0.66–2.53)
Staff members^a^: actively reaching out to patients that might postpone healthcare (yes)	42.3% Q4	52.9%	55.0%	36.8%	0.59 (0.19–1.89)	2.47* (1.22–7.94)	1.46 (0.49–4.33)
Staff members^a^: giving information or explanation about what the caregiver said to illiterate patients, patients with low health literacy, or migrants (yes)	64.6% Q2	70.6%	67.2%	62.7%	0.88 (0.26-3.00)	1.74 (0.83–3.64)	1.53 (0.48–4.85)
The availability of multilingual communication in the practice regarding…							
the practice answering machine (yes)	9.5% Q2	30.8%	3.5%	9.6%	11.81***(3.65–38.23)	2.86 (1.00-8.17)	4.12**(1.70-10.06)
the practice leaflet (yes)	13.1% Q2	50.0%	4.6%	12.2%	34.83***(5.70-212.9)	3.91 (0.82–18.66)	8.91*** (2.63–30.22)
the leaflet with information on COVID-19 (yes)	25.1% Q3	75.0%	8.0%	29.2%	26.14***(6.59–103.7)	3.82**(1.44–10.16)	6.84** (1.89–24.73)
the practice website (yes)	13.5% Q3	53.8%	8.5%	10.9%	11.44***(3.61–36.27)	1.24 (0.48–3.16)	9.24***(3.53–24.20)
The GP or GP trainee checked …							
the feasibility of isolation at home when indicated. (always)	32.1% Q1	26.1%	25.2%	36.6%	0.95 (0.43–2.09)	1.53 (0.93–2.52)	0.62 (0.29–1.30)
the feasibility of transport to another facility in case of a referral. (always)	43.4% Q2	39.5%	49.3%	41.0%	0.61 (0.30–1.25)	0.61* (0.39–0.97)	0.99 (0.50–1.98)
The GP or GP trainee screened whether a patient experienced …							
Domestic violence (more or much more than before COVID-19)	17.1% Q1	22.7%	17.4%	16.0%	1.22 (0.53–2.84)	0.74 (0.41–1.35)	1.65 (0.73–3.70)
financial problems (more or much more than before COVID-19)	40.9% Q1	50.0%	46.4%	36.2%	1.11 (0.55–2.24)	0.54* (0.34–0.87)	2.04 (1.03–4.02)
*Safe and effective care*							
Building/infrastructure of the practice							
Experiences of limitations to be able to provide high-quality care^b^. (to a large or limited extent)	55.5% Q2	53.2%	39.1%	64.6%	1.65 (0.80–3.41)	1.94** (1.21–3.10)	0.85 (0.43–1.70)
Considering making adjustments in future^b^. (to a large or limited extent)	37.1% Q1	39.1%	32.0%	39.8%	1.20 (0.57–2.51)	1.03 (0.64–1.68)	1.16 (0.58–2.32)
Appointment system							
Online appointment: informative message about symptoms patients may not enter the practice (yes)	81.5% Q1	66.7%	56.0%	89.2%	1.21 (0.38–3.81)	4.27*** (1.85–9.83)	0.28* (0.10–0.82)
Online appointment: patients needed to give a reason for encounter (yes)	68.2% Q2	59.1%	60.0%	71.2%	0.74 (0.25–2.15)	1.31 (0.62–2.79)	0.56 (0.22–1.44)
Appointment by phone: patients needed to give a reason for encounter (yes)	85.4% Q3	81.8%	84.4%	86.5%	0.81 (0.31–1.80)	1.07 (0.57–2.03)	0.75 (0.31–1.80)
Protocol for answering phone calls from potential COVID-19 patients							
Availability of a protocol (yes)	68.7% Q4	69.8%	61.8%	72.1%	1.26 (0.57–2.76)	1.11 (0.68–1.82)	1.13 (0.52–2.44)
Triage							
Using a protocol for answering calls if this was available. (always)	26.3% Q4	20.0%	29.1%	26.0%	0.56 (0.20–1.56)	0.69 (0.36–1.31)	0.81 (0.30–2.16)
Calling patients who made an online appointment to check infection risk. (always)	28.0% Q3	26.5%	21.8%	31.1%	0.94 (0.36–2.40)	1.44 (0.83–2.50)	0.65 (0.27–1.58)
Availability of a GP as a backup when a non-GP does the telephonic triage. (always)	79.8% Q2	55.0%	70.9%	86.3%	0.31* (0.10–0.94)	1.80 (0.85–3.80)	0.17** (0.06–0.51)
Availability of the most recent information on how to refer a patient to a triage station in each GP consultation room. (yes)	75.2% Q3	72.5%	75.4%	76.4%	0.88 (0.38–2.02)	1.09 (0.63–1.91)	0.81 (0.37–1.75)
Cleaning the practice							
Using a detailed cleaning protocol by cleaning employees during COVID-19.(always)	57.2% Q4	46.7%	58.3%	58.6%	0.50 (0.24–1.05)	0.98 (0.61–1.57)	0.52 (0.26–1.04)
Sufficient time between consultations for the disinfection. (always)	38.5% Q2	46.7%	41.4%	35.4%	1.18 (0.59–2.39)	0.83 (0.52–1.32)	1.43 (0.73–2.80)
Home visits							
Availability of a separate medical bag for (possible) infection-related consultations. (yes)	27.9% Q4	22.7%	36.3%	24.0%	0.47 (0.20–1.07)	0.66 (0.41–1.09)	0.70 (0.31–1.59)
*Timely care*							
Occurrence of a safety incident in which a patient with an urgent condition was seen late because							
the patient did not come to the practice sooner^b^. (yes)	70.9% Q4	61.9%	62.7%	76.5%	0.93 (0.44–1.99)	1.47 (0.88–2.46)	0.63 (0.30–1.33)
the patient did not know how to reach a GP^b^. (yes)	39.3% Q4	48.6%	22.8%	46.9%	2.58* (1.14–5.82)	2.63***(1.53–4.53)	0.98 (0.46–2.09)
the situation was assessed as non-urgent during the telephonic triage^b^. (yes)	26.0% Q4	18.4%	26.0%	26.9%	0.74 (0.29–1.90)	1.13 (0.65–1.98)	0.66 0.27–1.62)
Occurrence of safety incident because a patient with a fever caused by a non-COVID infection was seen late due to the COVID-19 protocol^b^. (yes)	47.9% Q4	46.3%	48.5%	48.0%	0.67 (0.32–1.42)	0.68 (0.42–1.11)	0.98 (0.48–2.01)
*Efficient care*							
Change of roles compared to before COVID-19 including a greater involvement of							
Staff members^a^: triaging of patients (yes)	91.1% Q1	88.9%	80.3%	95.2%	1.46 (0.27–7.98)	0.34 (0.11–1.02)	0.49 (0.08–2.90)
Staff members^a^: giving information and recommendations to patients contacting the practice by phone (yes)	85.4% Q3	88.9%	75.8%	88.5%	1.94 (0.35–10.65)	0.85 (0.34–2.11)	1.65 (0.29–9.21)

odds ratios (ORs) are used to represent the likelihood of outcomes. An OR > 1 indicates an increased likelihood of the outcome as the predictor variable increases, while an OR < 1 suggests a decreased likelihood. For ORs <1, interpretations can be inverted for clarity. This inversion aids in easier comprehension while maintaining statistical accuracy

Adjusted models included the following covariates: practice type (solo, duo, or group); being a teaching practice for GP trainees (yes or no); multidisciplinary of the practice (yes or no); and payment system of the practice (fee-for-service or capitation)

*p<0.05

**p<0.01

***p<0.001

^a^only GP practices with more than one paid staff member were included in the analyses

^b^these outcome variables were inverse scored to calculate the quartiles; Q1 included the eight countries that performed best on high-quality care for the respective outcome variable, and Q4 represented the eight countries that obtained the worse scored regarding high-quality care

## Discussion

The main aim of this paper was to describe the organization of care in Belgian GP practices during COVID-19 from the perspective of the six dimensions of quality of care: equity, patient-centeredness, safety, effectiveness, timeliness, and efficiency. Moreover, differences between the three Belgian regions were investigated, and Belgium was benchmarked against a pool of 36 other European countries. The results show that Belgian GP practices made important structural and organizational adjustments to guarantee high-quality care in all its dimensions but also encountered challenges in doing so.

Belgian GP practices made important efforts to deliver equitable and person-centered care, such as setting up outreach work. Proactive care for patients with a chronic condition was quite common in Belgium. However, practices did not frequently reach out to psychologically vulnerable patients or patients with known problems of domestic violence or issues related to raising or parenting a child. Based on the findings of another PRICOV-19 paper, the Belgian numbers were lower than the European average, except for proactive care for patients with a psychological vulnerability [30]. Setting up outreach work was one of the new tasks within practices during COVID-19. Half of the Belgian practices indicated that GPs and/or GP trainees were more often involved in actively reaching out to patients who might postpone healthcare, which was less the case for non-GP staff members. The importance of outreach work is highlighted by the high rates of postponement of care observed in recent population-based research during COVID-19, particularly among patients with a certain vulnerability [31]. Another international PRICOV-19 paper demonstrated that many GPs were happy with the overall task changes during COVID-19 but they also felt the need for further training [32]. Thus, training for GPs and non-GPs in particular to organize outreach work may be appropriate. Furthermore, a precondition for setting up proactive care is the identification of patients with a certain vulnerability. However, previous research demonstrated that person-related information is not systematically recorded or noted in the medical file of patients [33]. This paper also showed that Belgian practices did not have the habit of using the medical records to analyze practice performance or identify patients at risk. These findings stress the importance of integrating the patients’ social context in the medical record and using the medical record as a tool to guide practice policy, such as setting up initiatives for patients who seem to postpone healthcare [33].

In line with the international PRICOV-19 findings, the pandemic boosted the shift of tasks and roles from the GP to other staff members [32]. For example, during COVID-19, Belgian non-GPs were more engaged in informing or giving additional explanations about what the caregiver said to vulnerable patients. One vulnerable group was patients with limited knowledge of the official language. Although the survey did not specify whether multilingual communication included other official languages in Belgium (e.g., French in the FR) or minority languages (e.g., Arabic), the availability of multilingual communication varied between the Belgian regions in line with its official languages. Only a minority of the practices in the FR and WR had a leaflet, practice website, or message on their answering machine in multiple languages. Overall, language barriers are considered detrimental to the ability of patients to access care [22]. European countries face an increasing migrant population with diverse languages and cultural backgrounds, which will grow in the upcoming decennia [34, 35]. Therefore, other regions in Belgium and Europe can learn from practices in the BCR how to implement multilingual communication in the future.

Less than half of the practices always checked with patients about the feasibility of transport to another facility when being referred or isolated at home. Compared to before COVID-19, GPs and/or GP trainees screened patients’ financial status more often, but screening for domestic violence happened in less than one-fifth of practices. These findings raise concerns as more problems of financial distress and domestic violence have been observed worldwide since COVID-19 [36, 37]. The World Health Organization highlighted the critical role of PC in meeting the needs of vulnerable patients [38], but previous studies also reported that GPs would overestimate patients’ socioeconomic status [26]. Therefore, this study recommends preparing students as early as possible during their education in general practice to fulfil this key role as the primary contact point. The main topics could be the awareness of patient vulnerability and training them to discuss sensitive issues regarding the patient’s context. Further training is also warranted for GPs during their professional career to refine their skills.

Regarding safe and effective care, infection prevention and control (IPC) in processes and procedures should be prioritized in GP practices to avoid spreading the SARS-COV-2 virus among patients and staff members. Its importance ties in with the high rates of COVID-19 infections among healthcare professionals due to multiple prolonged exposures [39]. However, many Belgian practices experienced limitations related to the building or the practice’s infrastructure to provide safe care. Other studies refer to, for example, the inability to separate patient flows or insufficient air ventilation [40–42]. About one-third of Belgian practices felt that their building and infrastructure needed adjustment in the future, which is lower than the proportion overall found in participating counties in PRICOV-19 (i.e., approximately 54%) [43]. Moreover, Windak et al. indicated that among others, improved IPC equipment is an important factor associated with a reduced perceived need for infrastructural changes [43]. Therefore, IPC should be a point of attention in the conceptualization phase of a practice building. However, literature or recommendations on this topic are scarce. For example, the Dutch College of General Practitioners guidelines include IPC recommendations regarding personal hygiene, protective equipment, cleaning and disinfection of instruments, rooms, furniture, and objects, but not for the organization of the practice building [44]. Moreover, a manual on the (re)construction and design of multidisciplinary healthcare centers in the Netherlands only minimally focuses on how to design the building from an IPC perspective [45]. Also, IPC is barely addressed in the training of architects or designers. Initiatives to fill this knowledge gap are needed.

For most outcome variables on the appointment system and triage, Belgium was benchmarked in the middle against other European countries. However, Belgium ranked in the lowest quartile for always using a telephone protocol for potential COVID-patients, verifying the infection risk in uncertain cases, and providing separate medical bags for home visits involving potential infectious patients. Using protocols can contribute to a better quality of care and reduce patient safety incidents [16]. Therefore, professional organizations are encouraged to put IPC guidelines in the spotlight to reduce the risk of transmission of viruses.

This study found important shortcomings regarding the timeliness of care. Many Belgian practices were faced with patient safety incidents in which patients were seen too late because the patient did not know how to reach a GP, because the patient was wrongly assessed as non-urgent during the telephone triage, or the diagnosis was delayed because of the COVID-19 protocol. With these results, Belgian practices rank remarkably low in the European ranking. Nevertheless, these results are based on the GPs’ perceptions of whether these incidents happened. Consequently, the differences between countries could not only be explained by differences in patient safety but also differences in the patient safety culture between countries. In PC, a positive patient safety culture (PSC) manifests itself, among other things, in open communication and reporting and analysis of incidents in a non-punitive approach [46]. Sharp, Rannus, et al. [47] have already demonstrated that these elements may differ significantly from country to country among nurses in secondary care, implying an important influence of national culture on safety practices. Belgian practices may be associated with a positive PSC leading to a high number of reported incidents during COVID-19. To verify these statements, comparative cross-country research on the existing PSC in GP practices is essential. In any case, the pandemic resulted in the postponement of care and an increased risk of patient safety incidents [48, 49]. An awareness among GPs that this side effect occurs during major epidemics could be a starting point for practice improvement projects preparing the practice for future infectious outbreaks. These can be based on a critical incident analysis, the importance of which has been demonstrated previously [9, 50].

COVID-19 confronted GP practices with limited time availability, infrastructure, protective equipment, and even staff members due to COVID-19 infection or quarantine, which hampered efficient care delivery. Implementing protocols may also benefit the profitable use of resources and offer the possibility of delegating tasks among staff members. Belgian practices were aware of this as many duo and group practices reported that non-GPs took on a larger role in triaging patients and helping patients who contacted the practice. Practices were inundated by calls from patients, which rendered such support indispensable. These findings raised concerns about the situation and well-being of GPs working in solo practices. Analyses of the international dataset on PRICOV-19 confirmed higher distress among GPs in smaller practices [51]. Thus, research on how GPs working in small practices could be supported to deal with a high workload and improve efficiency is essential.

Adjusting for the structural practice characteristics in the regression analysis, limited significant differences were found between the three Belgian regions, indicating that the variations between the regions might stem from practice characteristics rather than cultural differences. This finding is in line with other studies showing the impact of several practice characteristics on quality of care outcomes. A national study in France demonstrated that multi-professional group practices are strongly related to a higher level of reorganization than other practice types during the pandemic [52]. A Dutch cross-sectional study in the pre-COVID era has pointed out that GP practices consisting of more than two GPs may have better safety management than small GP practices regarding medication, medical record keeping, and hygiene [53]. Literature on the impact of financing (capitation versus fee-for-service) during the pandemic is still inconsistent [15, 28], thus highlighting the need for further investigation.

In addition to practice characteristics, adaptations in PC during COVID-19 also vary among countries, according to previous research [54, 55]. This implies that characteristics of the healthcare system may play a part in coping with the challenges of COVID-19. However, cross-country comparative analyses on the international PRICOV-19 dataset, including the data from more than 5,000 GP practices across Europe, are needed to verify this hypothesis. These may focus on system and practice characteristics contributing to better quality care and the underlying mechanisms leading to this contribution.

### Strengths and limitations

Globally, experts have already stressed the lack of research on the position of PC during the COVID-19 pandemic [2, 9]. This study provided an answer regarding Belgium based on 479 GP practices. According to earlier studies in PC [52, 56], response rates of 25.1% and 81.3% for randomized and convenience sampling methods were reasonable for Belgium. The sample composition among the regions corresponded generally to the actual distribution of GPs in Belgium (IMA-AIM, 2021), implying that a representative sample participated in this study for this criterion. Other characteristics of the GP practices were not compared because of the lack of relevant comparative data. However, a few limitations should also be noted.

Firstly, participation in this study was voluntary, resulting in a risk of self-selection bias and a small sample size. Possibly, mainly GP practices interested in the quality of care and patient safety participated in the study. A few outcome variables had considerable missing data. However, the analysis did not address whether these missing values occurred randomly. Data were collected through an online self-reported survey, so no information on the actual practice organization is known. Furthermore, a few outcome variables focused on differences between the situation before and during COVID-19. Their results should be interpreted carefully as practices that already performed well could not make the same progress as other practices. In addition, only one survey was completed per GP practice as described in the study protocol, thanks to the close collaboration among the research teams involved. It implies that the reliability of the answers also relied on the familiarity of the participating staff member with the practice processes and procedures. However, the function of the participating staff member was not considered in the analyses. In addition, participants were mainly recruited through randomized sampling, supplemented by a convenient sample. However, to adhere to the General Data Protection Regulation (GDPR), the information concerning the sample allocation of each participant was removed during data processing, rendering it impossible to ascertain whether any significant discrepancies existed in the composition or performance between the two samples.

Data collection was spread between December 2020 and August 2021, owing to the time-consuming recruitment process. Due to the lack of an accurate registry of all GP practices in Belgium, recruitment had to be done at the GP level, which required looking up contact details and ensuring that only one GP was invited per practice. This period encompassed three large waves of the COVID-19 pandemic in Belgium, implying that the timing might have affected the study results. Therefore, the results only demonstrated a snapshot of the practice organization during COVID-19. Consequently, making any statements about possible permanent changes in Belgian practices’ practice organization or quality policy is impossible. Monitoring the amended practice organization and their sustainability over time might be useful.

## Conclusion

Overall, Belgian GP practices made important organizational efforts to deliver high-quality care in all six dimensions during COVID-19. Outreach work was organized for vulnerable patients regarding equitable and person-centered care. However, proactive care was more common for patients with a chronic condition than for patients with a psychological vulnerability or known problems of domestic violence or parenting situation. More than half of the practices were confronted with limitations regarding the infrastructure or building to deliver safe and effective care. In addition, safety incidents occurred in practices leading to delayed care among patients with an urgent condition. Many practices used the pandemic as an opportunity to enhance the efficiency of care by redistributing tasks and roles among GPs and non-GPs.

In terms of a leaflet with COVID-information, practice leaflet or website, or answering messages, the availability of multilingual communication varied between the Belgian regions in line with its official languages. Therefore, FR and WR practices may benefit from adopting strategies used in the BCR for effective multilingual communication in the future. Controlling for the structural practice characteristics in the regression analysis, limited significant differences were found between the three Belgian regions. Belgium performed relatively well on the European level, except for the outcome variables on the timeliness of care. Hereby, Belgian practices reported more incidents of delayed care among non-COVID-patients than in at least 28 other European countries. Future studies using different design methods are crucial to verify and elaborate on the conclusions here, particularly to understand which system and practice characteristics contribute to better quality care.

### Supplementary Information

**Additional file 1. **Survey questions and their original and recoded answer options that were the basis for the outcome variables, including the number of missing values per variable in the cleaned dataset.

**Additional file 2. **Comparison of Belgium to 36 other European countries and between the Belgian regions: descriptive statistics and unadjusted and adjusted binary logistic regression models for the six dimensions of quality of care.

## Data Availability

All data are centrally stored on the server of Ghent University (Belgium). All data was anonymized at Ghent University, and all raw data that could lead to the identification of the participants was permanently removed. Reasonable request is required to access non-identifiable data by users who are external to the PRICOV-19 consortium. Access will be subject to a data transfer agreement and following approval from the principal investigator of the PRICOV-19 study.

## Abbreviations

GP: General practitioner
GP practice: General practice
PC: Primary care
PRICOV-19: Quality and safety in PRImary care in times of COVid-19
BCR: Brussels-Capital Region
FR: Flemish Region
WR: Walloon Region
Q: quartile
IPC: Infection prevention and control
PSC: patient safety culture
SSMG: Société Scientifique de Médecine Générale
GDPR: General Data Protection Regulation
