# Supplementary material for: Quality of care in Belgian general practices during the COVID-19 pandemic: results of the cross-sectional PRICOV-19 study
Source: BMC Prim Care. 2024 Mar 5;24(Suppl 1):282. doi: 10.1186/s12875-024-02305-8 (PMC10916333; doi:10.1186/s12875-024-02305-8)
Supplement: Supplementary file 1 — Additional file 1. Survey questions and their original and recoded answer options that were the basis for the outcome variables, including the number of missing values per variable in the cleaned dataset. [file 12875_2024_2305_MOESM1_ESM.docx]

**Additional file 1: survey questions and their original and recoded answer options that were the basis for the outcome variables, including the number of missing values per variable in the cleaned dataset**

| Survey question | Original answer options | Recoded answer options | Missing values in the cleaned dataset (n, %) | |
| --- | --- | --- | --- | --- |
| *Person-centered and equitable care* | | |  | |
| *In this practice, one or more of the following initiatives were taken since the COVID-19 pandemic:* | | |  | |
| A list was compiled from the electronic medical record for at least one group of patients with a chronic disorder (e.g. all patients taking methotrexate and needing to be seen). |  Yes   No   I do not know |  No   Yes  Missing value: no answer, I do not know | 36/479 = 7.5% | |
| This practice contacted patients with a chronic condition who needed follow-up care. |  Yes   No   I do not know |  No   Yes  Missing value: no answer, I do not know | 26/479 = 5.4% | |
| This practice contacted psychologically vulnerable patients. |  Yes   No   I do not know |  No   Yes  Missing value: no answer, I do not know | 28/479 = 5.8% | |
| This practice contacted patients with previous problems of domestic violence or with a problematic parenting situation |  Yes   No   I do not know |  No   Yes  Missing value: no answer, I do not know | 57/479 = 11.9% | |
| Since the pandemic, the role of GPs or GP trainees might have changed. Please rate how much you agree with the following statement since the COVID-19 pandemic: | | |  | |
| Since the COVID-19 pandemic, GPs or GP trainees are more involved in actively reaching out to patients that might postpone healthcare. |  Strongly disagree   Disagree   Neutral   Agree   Strongly agree   I do not know/ not applicable |  Disagree or neutral opinion that the role is changed: strongly disagree, disagree, neutral   Agree that the role is changed: strongly agree, agree  Missing value: no answer; I do not know/ not applicable | 40 / 479 = 8.4% | |
| Since the pandemic, the role of non-GP staff members might have changed. Please rate how much you agree with the following statements since the COVID-19 pandemic? | | |  | |
| Since the COVID-19 pandemic, staff members are more involved in actively reaching out to patients that might postpone healthcare. |  Strongly disagree   Disagree   Neutral   Agree   Strongly agree   I do not know/ not applicable |  Disagree or neutral opinion that the role is changed: strongly disagree, disagree, neutral   Agree that the role is changed: strongly agree, agree  Missing value: no answer; I do not know/ not applicable | 92 / 333 = 27.6%^a^ | |
| Since the COVID-19 pandemic, staff members are more involved in giving information or explaining what a caregiver has said to illiterate patients, patients with low health literacy or migrants. |  Strongly disagree   Disagree   Neutral   Agree   Strongly agree   I do not know/ not applicable |  Disagree or neutral opinion that the role is changed: strongly disagree, disagree, neutral   Agree that the role is changed: strongly agree, agree  Missing value: no answer; I do not know/ not applicable | 96 / 333 = 28.8%^a^ | |
| Does the answering machine of this practice provide information in multiple languages? |  Yes, in multiple languages   No   I do not know   There is no answering machine |  Monolingual: no   Multilingual: yes, in multiple languages  Missing value: no answer, I do not know, there is no answering machine | 38 / 479 = 7.9% | |
| Is the leaflet of this practice available to patients in multiple languages? |  Yes, in multiple languages   No   I do not know   There is no practice leaflet |  Monolingual: no   Multilingual: yes, in multiple languages  Missing value: no answer, I do not know, there is no practice leaflet | 205 / 479 = 42.8% | |
| Does this practice have a leaflet with information on COVID-19 to give to patients? |  Yes, in one language   Yes, in multiple languages   No   I do not know |  Monolingual: yes, in one language   Multilingual: yes, in multiple languages  Missing value: no answer, I do not know, no | 245 / 479 = 52.6% | |
| Is the information on the website of this practice available in multiple languages? |  Yes, in multiple languages   No   I do not know   There is no website |  Monolingual: no   Multilingual: yes, in multiple languages  Missing value: no answer, I do not know, there is no website | 132 / 479 = 27.6% | |
| When a patient needs to isolate him/herself, the extent to which this is feasible at his/her home is checked with the patient. |  Never   Rarely   Sometimes   Usually   Always   I do not know |  Not always: never, rarely, sometimes, usually   Always  Missing value: no answer, I do not know | 13 / 479 = 2.7% | |
| When a patient is referred to another facility (e.g. the hospital, the triage station,…) it is checked whether he/she is able to go there. |  Never   Rarely   Sometimes   Usually   Always   I do not know |  Not always: never, rarely, sometimes, usually   Always  Missing value: no answer, I do not know | 19 / 479 = 4.0% | |
| To what extent have you checked with patients to determine if they (in)directly experienced domestic violence since the COVID-19 pandemic?  [This question is only presented to GPs and GP trainees] |  Not at all   Less than before   As much as before   More than before   Much more than before |  Not more than before COVID-19: not at all, less than before, as much as before   More than before COVID-19: more than before, much more than before  Missing value: no answer | 23 / 479 = 4.8% | |
| To what extent have you checked with patients to determine if they experienced financial problems since the COVID-19 pandemic?  [This question is only presented to GPs and GP trainees] |  Not at all   Less than before   As much as before   More than before   Much more than before |  Not more than before COVID-19: not at all, less than before, as much as before   More than before COVID-19: more than before, much more than before  Missing value: no answer | 25 / 479 = 5.2% | |
| *Safe and effective care* | | |  | |
| Since the COVID-19 pandemic, did you experience any limitations related to the building or the infrastructure of this practice to provide high-quality and safe care? |  To a large extent   To a limited extent   Hardly   None   I do not know |  No: none, hardly   Yes: to a large extent, to a limited extent  Missing value: no answer, I do not know | 1/479 = 0.2% | |
| Did the COVID-19 pandemic lead this practice to consider making adjustments in the future to the building or the infrastructure? |  To a large extent   To a limited extent   Hardly   None   I do not know |  No: none, hardly   Yes: to a large extent, to a limited extent  Missing value: no answer, I do not know | 6/479 = 1.3% | |
| Appointment system | | |  | |
| online appointment: showing informative message about  symptoms patients may not enter the practice |  Yes   No   I do not know   Not applicable |  No   Yes  Missing value: no answer, I do not know, not applicable | 205 / 479 = 42.8% | |
| online appointment: patients need to give a reason for  encounter |  Yes   No   I do not know |  No   Yes  Missing value: no answer, I do not know | 199 / 479 = 41.5% | |
| appointment by phone: patients need to give a reason for  encounter |  Yes   No   I do not know |  No   Yes  Missing value: no answer, I do not know | 27 / 479 = 5.6% | |
| Is a protocol been used in this practice when answering phone calls from potential COVID-19 patients? |  Yes, this protocol is based on a government guideline   Yes, this protocol is not based on a government guideline   No   I do not know |  There is no protocol: no   There is a protocol: yes, this protocol is based on a government guideline; yes, this protocol is not based on a government guideline  Missing value: no answer, I do not know | 23 / 479 = 4.8% | |
| Triage | | |  | |
| When answering these phone calls, how often this protocol used in this practice?  [This question is only presented to the practices with a protocol] |  Never   Rarely   Sometimes   Usually   Always   I do not know |  Not always: never, rarely, sometimes, usually   Always  Missing value: no answer, I do not know | 171 / 479 = 35.7% | |
| In the situation where telephonic triage is performed by someone other than a GP in this practice and he/she needs support when assessing a call, he/she can rely on support from a GP. |  Never   Rarely   Sometimes   Usually   Always   I do not know   Not applicable |  Not always: never, rarely, sometimes, usually   Always  Missing value: no answer, I do not know, not applicable | 198 / 479 = 41.3% | |
| In every GP consultation room in this practice, the most recent information on how to refer a patient to a triage station is immediately available (e.g. procedure, telephone numbers, which documents to provide). |  Yes, this information is available in print   Yes, this information is electronically available (e.g. on the computer desktop)   No, the GP can look for this information on a public website   Other   I do not know   Not applicable |  No: no, the GP can look for this information on a public website   Yes: yes, this information is available in print; Yes, this information is electronically available (e.g. on the computer desktop)  Missing value: no answer, other, I do not know, not applicable | 56 / 479 = 11.7% | |
| Sufficient time between consultations for the disinfection |  Never   Rarely   Sometimes   Usually   Always   I do not know/not applicable |  Not always: never, rarely, sometimes, usually   Always  Missing value: no answer, I do not know/not applicable | 18 / 479 = 3.8% | |
| Patients who made an appointment and where it is unclear whether they pose a risk of infection are called beforehand to verify this. |  Never   Rarely   Sometimes   Usually   Always   I do not know/not applicable |  Not always: never, rarely, sometimes, usually   Always  Missing value: no answer, I do not know/not applicable | 55 / 479 = 11.5% | |
| In the following question, we are interested whether the COVID-19 pandemic changed the application of the following infection prevention measures in this practice. | | |  | |
| When cleaning, the cleaning employees use a detailed protocol (e.g. what to clean, frequency, method) since COVID-19. |  Always   Sometimes   Never |  Not always: sometimes, never   Always  Missing value: no answer | 17 / 479 = 3.5% | |
| Home visits: availability of a separate medical bag for (possible) infection-related consultations since COVID-19. |  Always   Sometimes   Never |  Not always: sometimes, never   Always  Missing value: no answer | 14 / 479 = 2.9% | |
| *Timely care* | | | |  |
| Due to the complexity of primary care and the high degree of uncertainty, incidents can occur in all practices. Please indicate whether the following incidents occurred in this practice since the COVID-19 pandemic: | | |  | |
| A patient with an urgent condition was seen late because he/she did not come to the practice sooner. |  Yes   No   I do not know |  No   Yes  Missing value: no answer, I do not know | 56 /479 = 11.7% | |
| A patient with a serious condition was seen late because he/she did not know how to call on a GP. |  Yes   No   I do not know |  No   Yes  Missing value: no answer, I do not know | 43 / 479 = 9.0% | |
| A patient with an urgent condition was seen late, because the situation was assessed as non-urgent during the telephone triage. |  Yes   No   I do not know |  No   Yes  Missing value: no answer, I do not know | 95 / 479 = 19.8% | |
| A patient with a fever caused by an infection other than COVID-19 was seen late due to the fact the COVID-19 protocol was followed which delayed the care. |  Yes   No   I do not know |  No   Yes  Missing value: no answer, I do not know | 76 / 479 = 15.9% | |
| *Efficient care* | | |  | |
| Since the pandemic the role of staff members might have changed. Please rate how much you agree with the following statement since the COVID-19 pandemic: | | |  | |
| Staff members are more involved in the triage of patients (by phone, when entering the practice, …). |  Strongly disagree   Disagree   Neutral   Agree   Strongly agree   I do not know/ not applicable |  Disagree or neutral opinion that the role is changed: strongly disagree, disagree, neutral   Agree that the role is changed: strongly agree, agree  Missing value: no answer; I do not know/ not applicable | 85 / 333 = 25.5%^a^ | |
| Staff members are more involved in giving information and recommendations to patients contacting the practice by phone. |  Strongly disagree   Disagree   Neutral   Agree   Strongly agree   I do not know/ not applicable |  Disagree or neutral opinion that the role is changed: strongly disagree, disagree, neutral   Agree that the role is changed: strongly agree, agree  Missing value: no answer; I do not know/ not applicable | 87 / 333 = 26.1%^a^ | |

^a^only GP practices with more than one paid staff member were included in the analyses
