# Supplementary material for: Quality of care in Belgian general practices during the COVID-19 pandemic: results of the cross-sectional PRICOV-19 study
Source: BMC Prim Care. 2024 Mar 5;24(Suppl 1):282. doi: 10.1186/s12875-024-02305-8 (PMC10916333; doi:10.1186/s12875-024-02305-8)
Supplement: Supplementary file 2 — Additional file 2. Comparison of Belgium to 36 other European countries and between the Belgian regions: descriptive statistics and unadjusted and adjusted binary logistic regression models for the six dimensions of quality of care. [file 12875_2024_2305_MOESM2_ESM.docx]

**Additional File 2: Comparison of Belgium to 36 other European countries and between the Belgian regions:** **descriptive statistics and unadjusted and adjusted binary logistic regression models for the six dimensions of quality of care.**

|  | **Valid percentage** | | | | **Odds Ratio (95% Confidence Interval)** | | |
| --- | --- | --- | --- | --- | --- | --- | --- |
|  | **Belgium (total)**  **Quartile** | **Brussels-Capital Region**  **(BCR)** | **Walloon Region**  **(WR)** | **Flemish Region**  **(FR)** | **BCR vs. WR**  **Reference cat:**  **WR** | **FR vs. WR**  **Reference cat:**  **WR** | **BCR vs. FR**  **Reference cat:**  **FR** |
| *Person-centered and equitable care* | | | | | | | |
| The practice extracted a list of at least one group of patients with a chronic disorder from the electronic medical record system. (yes) | 19.6%  Q3 | 28.6% | 17.1% | 19.5% | 1.93 (0.87-4.31) | 1.17 (0.69-2.01) | 1.65 (0.79-3.44) |
|  |  |  |  |  | **1.62 (0.65-4.04)** | **1.05 (0.56-1.98)** | **1.54 (0.65-3.66)** |
| The practice actively reached out to … |  |  |  |  |  |  |  |
| patients with a chronic condition who needed follow-up care (yes) | 50.2%  Q3 | 55.8% | 43.7% | 52.6% | 1.63 (0.82-3.24) | 1.43 (0.95-2.16) | 1.14 (0.60-2.18) |
|  |  |  |  |  | **1.53 (0.72-3.25)** | **1.03 (0.64-1.67)** | **1.48 (0.72-3.05)** |
| psychologically vulnerable patients (yes) | 35.6%  Q2 | 37.8% | 31.9% | 37% | 1.30 (0.64-2.61)  **1.02 (0.46-2.24)** | 1.25 (0.81-1.93)  **0.81 (0.48-1.48)** | 1.04 (0.54-1.99)  **1.26 (0.60-2.66)** |
| patients with known problems of domestic violence or families with a known  problematic parenting situation (yes) | 14.9%  Q2 | 27.5% | 10.2% | 15.5% | 3.33**(1.37-8.09) | 1.61 (0.84-3.10) | 2.07 (0.95-4.49) |
|  |  |  |  |  | **2.78* (1.04-7.41)** | **1.15 (0.55-2.40)** | **2.42 (0.99-5.90)** |
| Change of roles compared to before COVID-19, including a greater involvement of… |  |  |  |  |  |  |  |
| GP or GP trainees: actively reaching out to patients that might postpone healthcare  (yes) | 50.2%  Q2 | 53.3% | 56.8% | 45.6% | 0.87 (0.44-1.70) | 0.64* (0.42-0.96) | 1.37 (0.72-2.58) |
|  |  |  |  |  | **0.84 (0.42-1.69)** | **0.65 (0.41-1.04)** | **1.29 (0.66-2.53)** |
| staff members^a^: actively reaching out to patients that might postpone healthcare  (yes) | 42.3%  Q4 | 52.9% | 55.0% | 36.8% | 0.92 (0.31-2.71) | 2.10* (1.15-3.82) | 1.93 (0.71-5.27) |
|  |  |  |  |  | **0.59 (0.19-1.89)** | **2.47* (1.22-7.94)** | **1.46 (0.49-4.33)** |
| staff members^a^: giving information or explanation about what the caregiver said to  illiterate patients, patients with low health literacy, or migrants (yes) | 64.6%  Q2 | 70.6% | 67.2% | 62.7% | 1.17 (0.36-3.78) | 1.22 (0.65-2.28) | 1.43 (0.48-4.26) |
|  |  |  |  |  | **0.88 (0.26-3.00)** | **1.74 (0.83-3.64)** | **1.53 (0.48-4.85)** |
| The availability of multilingual communication in the practice regarding… |  |  |  |  |  |  |  |
| the practice answering machine. (yes) | 9.5%  Q2 | 30.8% | 3.5% | 9.6% | 12.18***(3.97-37.40) | 2.92* (1.09-7.79) | 4.12***(1.89-9.25) |
|  |  |  |  |  | **11.81***(3.65-38.23)** | **2.86 (1.00-8.17)** | **4.12**(1.70-10.06)** |
| the practice leaflet. (yes) | 13.1%  Q2 | 50.0% | 4.6% | 12.2% | 20,67***(4.83-88.36) | 2.86 (0.83-9.88) | 7.22***(2.71-19.21) |
|  |  |  |  |  | **34.83***(5.70-212.9)** | **3.91 (0.82-18.66)** | **8.91*** (2.63-30.22)** |
| the leaflet with information on COVID-19. (yes) | 25.1%  Q3 | 75.0% | 8.0% | 29.2% | 34.29***(9.60-122.5) | 4.71***(1.98-11.20) | 7.29***(2.46-21.58) |
|  |  |  |  |  | **26.14***(6.59-103.7)** | **3.82**(1.44-10.16)** | **6.84** (1.89-24.73)** |
| the practice website. (yes) | 13.5%  Q3 | 53.8% | 8.5% | 10.9% | 12.50***(4.19-37.29) | 1.31 (0.55-3.14) | 9.56***(4.00-22.86) |
|  |  |  |  |  | **11.44***(3.61-36.27)** | **1.24 (0.48-3.16)** | **9.24***(3.53-24.20)** |
| The GP or GP trainee always checked … |  |  |  |  |  |  |  |
| the feasibility of isolation at home when indicated. (always) | 32.1% | 26.1% | 25.2% | 36.6% | 1.05 (0.49-2.24) | 1.72* (1.10-2.69) | 0.61 (0.30-1.23) |
|  | Q1 |  |  |  | **0.95 (0.43-2.09)** | **1.53 (0.93-2.52)** | **0.62 (0.29-1.30)** |
| the feasibility of transport to another facility in case of a referral. (always) | 43.4%  Q2 | 39.5% | 49.3% | 41.0% | 0.67 (0.34-1.35) | 0.72 (0.48-1.07) | 0.94 (0.49-1.81) |
|  |  |  |  |  | **0.61 (0.30-1.25)** | **0.61* (0.39-0.97)** | **0.99 (0.50-1.98)** |
| The GP or GP trainee screened whether a patient experienced … |  |  |  |  |  |  |  |
| Domestic violence. (more or much more than before COVID-19) | 17.1%  Q1 | 22.7% | 17.4% | 16.0% | 1.40 (0.61-3.20) | 0.91 (0.53-1.56) | 1.54 (0.71-3.35) |
|  |  |  |  |  | **1.22 (0.53-2.84)** | **0.74 (0.41-1.35)** | **1.65 (0.73-3.70)** |
| financial problems. (more or much more than before COVID-19) | 40.9%  Q1 | 50.0% | 46.4% | 36.2% | 1.15 (0.59-2.27) | 0.65* (0.43-0.99) | 1.76 (0.93-3.35) |
|  |  |  |  |  | **1.11 (0.55-2.24)** | **0.54* (0.34-0.87)** | **2.04 (1.03-4.02)** |
| *Safe and effective care* |  |  |  |  |  |  |  |
| Building/infrastructure of the practice |  |  |  |  |  |  |  |
| Experiences of limitations to be able to provide high-quality care^b^. (to a large or  limited extent) | 55.5%  Q2 | 53.2% | 39.1% | 64.6% | 1.77 (0.92-3.43) | 2.85***(1.90-4.29) | 0.62 (0.33-1.16) |
|  |  |  |  |  | **1.65 (0.80-3.41)** | **1.94** (1.21-3.10)** | **0.85 (0.43-1.70)** |
| Considering adjustments in future^b^. (to a large or limited extent) | 37.1%  Q1 | 39.1% | 32.0% | 39.8% | 1.37 (0.69-2.71) | 1.40 (0.92-2.13) | 0.98 (0.52-1.85) |
|  |  |  |  |  | **1.20 (0.57-2.51)** | **1.03 (0.64-1.68)** | **1.16 (0.58-2.32)** |
| Appointment system |  |  |  |  |  |  |  |
| Online appointment: informative message about symptoms patients may not enter  the practice (yes) | 81.5%  Q1 | 66.7% | 56.0% | 89.2% | 1.57 (0.54-4.56) | 6.46***(3.17-13.18) | 0.24** (0.09-0.67) |
|  |  |  |  |  | **1.21 (0.38-3.81)** | **4.27*** (1.85-9.83)** | **0.28* (0.10-0.82)** |
| Online appointment: patients needed to give a reason for encounter (yes) | 68.2%  Q2 | 59.1% | 60.0% | 71.2% | 0.96 (0.35-2.67) | 1.64 (0.87-3.12) | 0.59 (0.24-1.44) |
|  |  |  |  |  | **0.74 (0.25-2.15)** | **1.31 (0.62-2.79)** | **0.56 (0.22-1.44)** |
| Appointment by phone: patients needed to give a reason for encounter (yes) | 85.4%  Q3 | 81.8% | 84.4% | 86.5% | 0.83 (0.34-2.03) | 1.19 (0.67-2.11) | 0.70 (0.30-1.63) |
|  |  |  |  |  | **0.81 (0.31-1.80)** | **1.07 (0.57-2.03)** | **0.75 (0.31-1.80)** |
| Protocol for answering phone calls from potential COVID-19 patients |  |  |  |  |  |  |  |
| Availability of a protocol. (yes) | 68.7%  Q4 | 69.8% | 61.8% | 72.1% | 1.43 (0.69-2.97) | 1.60* (1.04-2.46) | 0.89 (0.44-1.80) |
|  |  |  |  |  | **1.26 (0.57-2.76)** | **1.11 (0.68-1.82)** | **1.13 (0.52-2.44)** |
| Triage |  |  |  |  |  |  |  |
| Using a protocol for answering calls if this was available. (always) | 26.3%  Q4 | 20.0% | 29.1% | 26.0% | 0.61 (0.22-1.67) | 0.86 (0.49-1.51) | 0.71 (0.27-1.84) |
|  |  |  |  |  | **0.56 (0.20-1.56)** | **0.69 (0.36-1.31)** | **0.81 (0.30-2.16)** |
| Calling patients who made an online appointment to check infection risk. (always) | 28.0%  Q3 | 26.5% | 21.8% | 31.1% | 1.29 (0.54-3.07) | 1.62 (0.99-2.64) | 0.80 (0.36-1.78) |
|  |  |  |  |  | **0.94 (0.36-2.40)** | **1.44 (0.83-2.50)** | **0.65 (0.27-1.58)** |
| Availability of a GP as a backup when a non-GP did the telephonic triage. (always) | 79.8%  Q2 | 55.0% | 70.9% | 86.3% | 0.50 (0.18-1.37) | 2.58** (1.36-4.91) | 0.20**(0.07-0.52) |
|  |  |  |  |  | **0.31* (0.10-0.94)** | **1.80 (0.85-3.80)** | **0.17** (0.06-0.51)** |
| Availability of the most recent information on how to refer a patient to a triage  station in each GP consultation room. (yes) | 75.2%  Q3 | 72.5% | 75.4% | 76.4% | 0.81 (0.36-1.83) | 0.95 (0.57-1.56) | 0.86 (0.41-1.82) |
|  |  |  |  |  | **0.88 (0.38-2.02)** | **1.09 (0.63-1.91)** | **0.81 (0.37-1.75)** |

| Cleaning the practice |  |  |  |  |  |  |  |
| --- | --- | --- | --- | --- | --- | --- | --- |

| Sufficient time between consultations for the disinfection. (always) | 38.5%  Q2 | 46.7% | 41.4% | 35.4% | 1.24 (0.63-2.43) | 0.78 (0.51-1.18) | 1.60 (0.84-3.14) |
| --- | --- | --- | --- | --- | --- | --- | --- |
|  |  |  |  |  | **1.18 (0.59-2.39)** | **0.83 (0.52-1.32)** | **1.43 (0.73-2.80)** |
| Using a detailed cleaning protocol by cleaning employees during COVID-19.(always) | 57.2%  Q4 | 46.7% | 58.3% | 58.6% | 0.63 (0.32-1.23) | 1.01 (0.92-1.52) | 0.62 (0.33-1.16) |
|  |  |  |  |  | **0.50 (0.24-1.05)** | **0.98 (0.61-1.57)** | **0.52 (0.26-1.04)** |
| Home visits |  |  |  |  |  |  |  |
| Availability of a separate medical bag for (possible) infection-related consultations.  (yes) | 27.9%  Q4 | 22.7% | 36.3% | 24.0% | 0.52 (0.24-1.13) | 0.55** (0.36-0.86) | 0.93 (0.44-1.99) |
|  |  |  |  |  | **0.47 (0.20-1.07)** | **0.66 (0.41-1.09)** | **0.70 (0.31-1.59)** |
| *Timely care* | | | | | | |  |
| Occurrence of a safety incident in which a patient with an urgent condition was seen late because … |  |  |  |  |  |  |  |
| the patient did not come to the practice sooner^b^. (yes) | 70.9%  Q4 | 61.9% | 62.7% | 76.5% | 0.97 (0.47-1.98) | 1.94** (1.24-3.05) | 0.50*(0.25-0.99) |
|  |  |  |  |  | **0.93 (0.44-1.99)** | **1.47 (0.88-2.46)** | **0.63 (0.30-1.33)** |
| the patient did not know how to reach a GP^b^. (yes) | 39.3%  Q4 | 48.6% | 22.8% | 46.9% | 3.20** (1.46-7.03) | 3.00***(1.83-4.92) | 1.07 (0.52-2.18) |
|  |  |  |  |  | **2.58* (1.14-5.82)** | **2.63***(1.53-4.53)** | **0.98 (0.46-2.09)** |
| the situation was assessed as non-urgent during the telephonic triage^b^. (yes) | 26.0%  Q4 | 18.4% | 26.0% | 26.9% | 0.64 (0.26-1.60) | 1.04 (0.64-1.71) | 0.62 (0.26-1.47) |
|  |  |  |  |  | **0.74 (0.29-1.90)** | **1.13 (0.65-1.98)** | **0.66 0.27-1.62)** |
| Occurrence of safety incident because a patient with a fever caused by a non-COVID infection was seen late due to the COVID-19 protocol^b^. (yes) | 47.9%  Q4 | 46.3% | 48.5% | 48.0% | 0.92 (0.46-1.85) | 0.98 (0.64-1.49) | 0.94 (0.48-1.82) |
|  |  |  |  |  | **0.67 (0.32-1.42)** | **0.68 (0.42-1.11)** | **0.98 (0.48-2.01)** |
| *Efficient care* |  |  |  |  |  |  |  |
| Change of roles compared to before COVID-19, including a greater involvement of… |  |  |  |  |  |  |  |
| staff members^a^: triaging patients (yes) | 91.1%  Q1 | 88.9% | 80.3% | 95.2% | 1.96 (0.40-9.70) | 0.20**(0.08-0.53) | 0.40 (0.08-2.05) |
|  |  |  |  |  | **1.46 (0.27-7.98)** | **0.34 (0.11-1.02)** | **0.49 (0.08-2.90)** |
| staff members^a^: giving information and recommendations to patients contacting the  practice by phone (yes) | 85.4 %  Q3 | 88.9% | 75.8% | 88.5% | 2.55 (1.16-5.21) | 0.41* (0.19-0.87) | 1.04 (0.22-4.88) |
|  |  |  |  |  | **1.94 (0.35-10.65)** | **0.85 (0.34-2.11)** | **1.65 (0.29-9.21)** |

Adjusted models are shown in **bold**, including the following covariates: practice type (solo, duo, or group); being a teaching practice for GP trainees (yes or no); multidisciplinary of the practice (yes or no); and payment system of the practice (fee-for-service or capitation); ^a^only GP practices with more than one paid staff member were included in the analyses; ^b^these outcome variables were inverse scored to calculate the quartiles; Q1 included the eight countries that performed best on high-quality care for the respective outcome variable, and Q4 represented the eight countries that obtained the worse scored regarding high-quality care; *p<0.05; **p<0.01; ***p<0.001

Note: odds ratios (ORs) are used to represent the likelihood of outcomes. An OR > 1 indicates an increased likelihood of the outcome as the predictor variable increases, while an OR < 1 suggests a decreased likelihood. For ORs <1, interpretations can be inverted for clarity. This inversion aids in easier comprehension while maintaining statistical accuracy.
